# Therapeutic strategies in an outbreak scenario to treat the novel coronavirus originating in Wuhan, China

**DOI:** 10.12688/f1000research.22211.2

**Published:** 2020-02-07

**Authors:** Robert L. Kruse

**Affiliations:** 1 Department of Pathology, Johns Hopkins Hospital, Baltimore, Maryland, 21287, USA

**Keywords:** coronavirus, Wuhan, neutralizing antibody, ACE2, outbreak, 2019-nCoV

## Abstract

A novel coronavirus (2019-nCoV) originating in Wuhan, China presents a potential respiratory viral pandemic to the world population. Current efforts are focused on containment and quarantine of infected individuals. Ultimately, the outbreak could be controlled with a protective vaccine to prevent 2019-nCoV infection. While vaccine research should be pursued intensely, there exists today no therapy to treat 2019-nCoV upon infection, despite an urgent need to find options to help these patients and preclude potential death. Herein, I review the potential options to treat 2019-nCoV in patients, with an emphasis on the necessity for speed and timeliness in developing new and effective therapies in this outbreak. I consider the options of drug repurposing, developing neutralizing monoclonal antibody therapy, and an oligonucleotide strategy targeting the viral RNA genome, emphasizing the promise and pitfalls of these approaches. Finally, I advocate for the fastest strategy to develop a treatment now, which could be resistant to any mutations the virus may have in the future. The proposal is a biologic that blocks 2019-nCoV entry using a soluble version of the viral receptor, angiotensin-converting enzyme 2 (ACE2), fused to an immunoglobulin Fc domain (ACE2-Fc), providing a neutralizing antibody with maximal breath to avoid any viral escape, while also helping to recruit the immune system to build lasting immunity. The ACE2-Fc therapy would also supplement decreased ACE2 levels in the lungs during infection, thereby directly treating acute respiratory distress pathophysiology as a third mechanism of action. The sequence of the ACE2-Fc protein is provided to investigators, allowing its possible use in recombinant protein expression systems to start producing drug today to treat patients under compassionate use, while formal clinical trials are later undertaken. Such a treatment could help infected patients before a protective vaccine is developed and widely available in the coming months to year(s).

## Introduction

A mysterious illness causing pneumonia in December 2019 in Wuhan, China is now growing into a potential pandemic. These pneumonia cases were eventually characterized to be caused by a novel coronavirus (2019-nCoV) ^1^, of which Severe Acute Respiratory Syndrome (SARS) ^2^ and Middle East Respiratory Syndrome (MERS) ^3^ are members. SARS and MERS famously caused their own outbreak concerns when they were originally identified. SARS caused significant economic damage to Hong Kong and Southern China, before spreading to other countries. Ultimately, SARS infected up to 8,098 people and caused 774 deaths according to the World Health Organization (WHO) ^4^.

The novel coronavirus, 2019-nCoV, is now quickly spreading across the world after originating in Wuhan ^1^. Human-to-human transmission of 2019-nCoV has been confirmed in familial case cluster reports ^5^, as additional cases continue to be identified in different cities in China and countries around the world. Clinical symptoms of 2019-nCoV infection include fever, cough, and myalgia or fatigue with pneumonia demonstrated on chest CT scan imaging ^6^. Within China, the city of Wuhan along with several others has been shut down, with individuals not allowed to leave the city in an effort to contain the virus; such efforts are largely unprecedented in a city of this size ( https://www.nytimes.com/2020/01/22/world/asia/coronavirus-quarantines-history.html). For now, many travelers are being screened for fever (≥38°C) and reported recent history of travel to Wuhan in order to triage diagnostic testing ^7^.

These efforts resemble not only what happened with SARS in 2002–2003, but also the Ebola virus outbreak in 2014–2015. During those outbreaks, special protocols were put in place to quarantine any infected individuals and identify patient contacts at risk ^8^. Healthcare workers were also at risk, and despite extensive personal protective equipment measures, clinical providers did get infected in both outbreaks ^9^. There were no specific, antiviral treatments for SARS or Ebola at the time of the outbreaks beyond supportive measures ^10, 11^, which is a similar situation that healthcare systems are facing with 2019-nCoV.

The dire situations facing patients in outbreak scenarios demand quick responses by the healthcare community and the biotech industry. Unfortunately, many of the traditional options that guide drug development are inadequate for outbreaks; a process that takes years can’t help patients who are dying today, and economies that are being halted. In these situations, studies have often been conducted on compassionate use, and clinical trial approvals expedited. This was most recently seen in the 2014–2015 Ebola outbreak, where a variety of clinical trial candidates were studied. Many of these therapies failed, but ultimately a vaccine did emerge that was fully protective against the virus ^12^. It is important to note that, unlike the current situation with 2019-nCoV, Ebola had already been studied for years and this particular neutralizing vaccine made and tested in preclinical animal models years prior to the outbreak ^13^. For 2019-nCoV, beyond knowing the sequence of spike (S) protein of the coronavirus (GenBank: MN908947.3), there are no studies on how immunogenic this particular protein will be beyond surrogate comparisons to SARS and MERS, which limits the potential ability to quickly produce a vaccine. Moreover, while a vaccine would be greatly effective in helping to stop the spread of 2019-nCoV, an effective therapy is also needed for the patients infected with 2019-nCoV today, similar to the situation of Ebola patients needing effective therapies while vaccines were being developed.

In this article, I will outline different potential treatment options that could be pursued as a therapy for 2019-nCoV virus, keeping the focus on agents that could be rapidly tested in patients today and broadly effective in spite of limited knowledge of the biology of 2019-nCoV. Simply stated, there is limited time for basic studies of 2019-nCoV in research labs, while patients need effective therapies today. I finally propose the best potential treatment option in my opinion, along with instructions on how to manufacture the therapy for testing in patients today.

## Treatment strategies against 2019-nCoV

### Developing neutralizing antibodies to 2019-nCoV

Coronavirus entry starts with the S protein binding to a target receptor on the cell surface, where after fusion is mediated at the cell membrane, delivering the viral nucleocapsid inside the cell for subsequent replication ^14^. The S protein is famous for causing syncytial formation between infected cells and other receptor-bearing cells around them, emphasizing that the S protein does not function in just the virion state alone.

A neutralizing antibody targeting the S protein on the surface of 2019-nCoV is likely the first therapy contemplated by biomedical researchers in academia and industry, providing passive immunity to disease ^15^. The recently published genome sequence of 2019-nCoV (GenBank: MN908947.3) allows researchers to perform gene synthesis in the lab and consider expressing the S protein as an immunogen. Traditional methods of screening mice or rabbits for neutralizing antibodies may be too slow for this outbreak, but faster methods such as using phage or yeast display libraries that express antibody fragments could be used quickly to identify lead candidates for viral neutralization ^16, 17^. The challenge is that any antibody candidate would need to be rigorously tested in cell culture and animal models to confirm that it can neutralize 2019-nCoV and prevent infection. Furthermore, several isolates would need to be tested that are circulating in the population to try to assess if sufficient breadth of coverage is obtained with the neutralizing antibody. Information from other coronaviruses species like SARS would be helpful as to where to target the best epitope in order to produce neutralizing antibodies (the receptor-binding domain in the S protein is a key target) ^18^, but again this is a slow and challenging process, which may not yield significant gains for several months. Moreover, ultimately a cocktail of antibodies may be required to ensure full protection for patients, which would add additional complexity for formulation and manufacturing. Like some of the therapeutic options discussed below, the ability to express any lead candidates in lower organisms for protein expression (bacteria, yeast, insect cells) would facilitate faster production of therapy for patients ^19^.

An alternative strategy of generating neutralizing antibodies against 2019-nCoV S protein would be to immunize large animals (sheep, goat, cow) with the 2019-nCoV S protein, and then purifying polyclonal antibodies from the animals ^20^. This strategy may serve an expedited service in the setting of an outbreak and has many advantages such as simplifying production and manufacturing, but has limited guarantees that each animal would produce neutralizing antisera, or what the antibody titer would be in each animal ^21^. Moreover, there is also the human immune response against foreign immunoglobulins to other species, which would potentially complicate any treatment scenarios ^22^. In a truly desperate scenario, this strategy may be viable for a short-term, but would not easily scale in the 2019-nCoV outbreak, which is already rapidly multiplying.

### Using oligonucleotides against 2019-nCoV RNA genome

Beyond targeting the surface proteins of 2019-nCoV, one could also target the RNA genome itself for degradation. This RNA genome sequence of 2019-nCoV was recently published (GenBank: MN908947.3), and one strategy that could be considered then, is the use of small interfering RNA (siRNA) or antisense oligonucleotides (ASO) to combat the virus by targeting its RNA genome ^23^. The challenge with this strategy is multi-fold. First, the conserved RNA sequence domains of CoV-2019 are not known. Identifying conserved sequences is essential in order to optimize siRNA targeting and avoid viral escape of the oligonucleotide strategy. One could look at genome homology of 2019-nCoV to the SARS virus for comparison of conserved sequences, but this would still be guesswork. A second challenge is how the oligonucleotides would be delivered into the lungs. Advances have been made into delivery vehicles such as lipid nanoparticles that can mediate some delivery into the lungs ^24^. It is unknown, however, if enough siRNA’s or ASO’s would be effectively delivered within the lungs to stop the infection or make a difference in its clinical course. For example, if 25% of alveolar epithelial cells in the lung had siRNA or ASO in them, that efficiency might be a great success for traditional gene therapy, but would hardly make any difference in a viral infection. Such an explanation is also likely why siRNA candidates against Ebola failed in trials ^25^, despite significant success in preclinical animal models ^26, 27^. Lastly, even if one assumed that siRNA was effective clinically, there is a limited ability to scale up manufacturing of siRNA drugs to a large infected population. Current siRNA and ASO therapies are manufactured for rare diseases, and there are no available resources existing to manufacture the medications quickly.

### Repurposing currently available antiviral medications

Ideal agents to fight 2019-nCoV would be approved small molecule drugs that could inhibit different aspects of the viral life cycle, ultimately inhibiting replication. Two classes of potential targets are viral polymerases ^28^ and protease inhibitors ^29^, both of which are components of human immunodeficiency virus (HIV) and hepatitis C virus (HCV) antiviral regimens. Pilot clinical studies are already ensuing by desperate clinicians with various repurposed antiviral medicines. This has been done in every viral outbreak previously with limited success, outside of case reports ^30^. Indeed, during the Ebola outbreak, none of the repurposed small molecule drugs were definitively shown to improve the clinical course across all patients ^31^. The 2019-nCoV could be different, and there are initial positive reports that lopinavir and ritonavir, which are HIV protease inhibitors, have some clinical efficacy against 2019-nCoV, similar to prior studies using them against SARS ^32^. Research should continue to be undertaken to screen other clinically available antivirals in cell culture models of 2019-nCoV, in hopes that a drug candidate would emerge useful against the virus that could be rapidly implemented in the clinic. One promising example could be remdesivir, which interferes with the viral polymerase and has shown efficacy against MERS in mouse models ^33^. For further information, reviews of previous drug repurposing efforts for coronaviruses are provided ^34, 35^. Though these repurposed medications may hold promise, it is still reasonable to pursue novel, 2019-nCoV specific therapies to complement potential repurposed drug candidates.

### Passive antibody transfer from convalescent patient sera

A simple but potentially very effective tool that can be used in infectious outbreaks is to use the serum of patients who have recovered from the virus to treat patients who contract the virus in the future ^36^. Patients with resolved viral infection will develop a polyclonal antibody immune response to different viral antigens of 2019-nCoV. Some of these polyclonal antibodies will likely neutralize the virus and prevent new rounds of infection, and the patients with resolved infection should produce 2019-nCoV antibodies in high titer.

Patients with resolved cases of 2019-nCoV can simply donate plasma, and then this plasma can be transfused into infected patients ^37^. Given that plasma donation is well established, and the transfusion of plasma is also routine medical care, this proposal does not need any new science or medical approvals in order to be put into place. Indeed, the same rationale was used in the treatment of several Ebola patients with convalescent serum during the outbreak in 2014–2015, including two American healthcare workers who became infected ^38^.

As the outbreak continues, more patients who survived infection will become available to serve as donors to make antisera for 2019-nCoV, and a sizeable stock of antisera could be developed to serve as a treatment for the sickest patients. Unfortunately, the exponential growth of the outbreak would work against this strategy, since the growing number of cases would likely outstrip the ability of previous patients to provide donor plasma as treatment. Moreover, convalescent patient sera would have significant variability in the potency of antiviral effect, making it less ideal ^37^. While transfusion medicine services should certainly pursue convalescent patient sera as an option right now for patient treatment, it is ultimately limited in its effective scope of controlling the outbreak.

## Proposal for new 2019-nCoV therapies

The simplest and most direct approach to combating 2019-nCoV during the outbreak would be one to neutralize the virus from entering cells, the function that antibodies normally perform in the body ^39^. For the reasons mentioned above when discussing neutralizing antibodies, it will be difficult to validate a broadly neutralizing antibody quickly, and a challenge to make sure that the mutating RNA virus will not escape its neutralization. A cocktail antibody approach could be undertaken as was explored to treat the Ebola pandemic ^40^, but would add complexity to the manufacturing process.

However, there is another strategy to pursue in this scenario that does not rely on targeting the viral glycoprotein directly. In this strategy, a neutralizing effect could be obtained by targeting the viral receptor protein on the cell surface, thereby blocking the virus from binding to it and gaining entry. Fortunately, scientists have already uncovered the identity of the viral receptor in cell culture. A recent pre-print publication found that the 2019-nCoV uses the angiotensin-converting enzyme 2 (ACE2) as a receptor for cell entry ^41^, which is the same receptor that the SARS coronavirus uses for entry ^42^. For both viruses, the coronavirus binds to ACE2 through its S protein on the virion, where after fusion of the viral membrane and cell membrane will occur. Subsequently, the RNA virus will replicate its genome inside the cell, and ultimately make new virions that will be secreted to infect other cells. The coincidence of SARS and 2019-nCoV using ACE2 receptor opens up the possibility of using the extensive research studied on SARS entry and applying it to 2019-nCoV. Based on the SARS literature, several potential blocking strategies could be considered, which were shown to be effective in preventing infection in SARS models.

### Blocking agents that bind to ACE2 receptor

The first strategy would consist of administering to patients an agent that would bind to ACE2. The key advantage here is that the host ACE2 protein will not change, so there is no concern about escape from binding the therapeutic agent. Moreover, the virus will not have the ability to mutate and bind an entirely new host receptor in the time frame of this outbreak; such functional relationships are established by evolution over long periods. By analogy, the influenza virus changes the mutations on its surface to escape antibody neutralization every year, but it always focuses on using sialic acid on the cell surface as an entry receptor ^43^.

**Figure 1.**
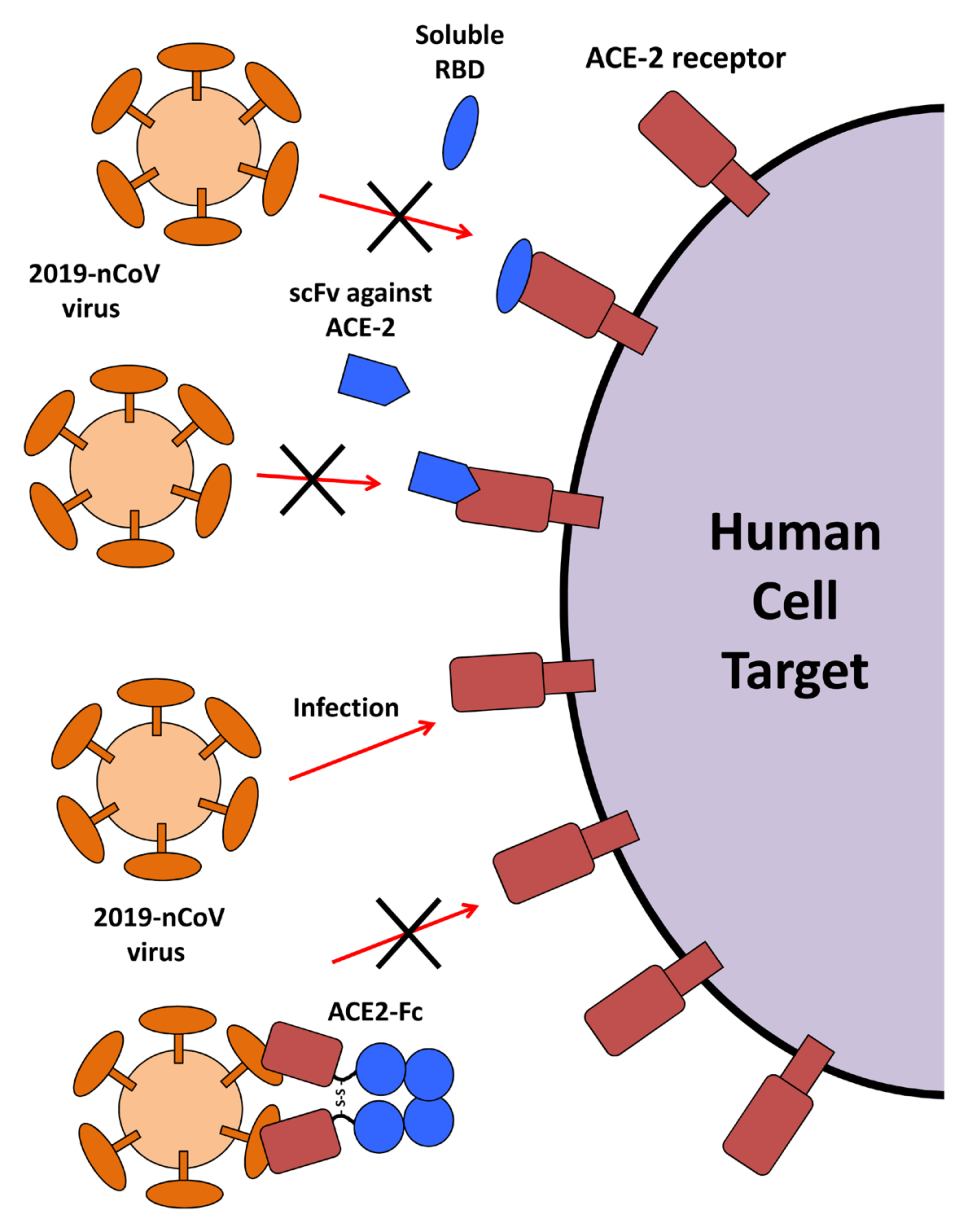
Therapeutic agents that could be used to block 2019-nCoV from infecting cells. Target cells expressing ACE2 include lung and gastrointestinal tissues in the human body. The large spike protein on the surface of the coronavirus binds to ACE2 on infected cells, leading to cell entry. Three proposed strategies would block this interaction would abrogate infection. In the first, the receptor-binding domain (RBD) of the spike protein from SARS or 2019-nCoV would be administered, thereby binding ACE2 and saturating available sites. Alternatively, an antibody or single chain antibody fragment (scFv) could be administered against ACE2 to accomplish the same. A third strategy would target the coronavirus virions directly by using the ACE2 extracellular domain as bait to bind to spike protein. An Fc domain fused to ACE2 would facilitate prolonged circulation of the biologic (ACE2-Fc).

There are two known options for agents to bind to ACE2. The first is using the small receptor-binding domain (RBD) from the SARS S protein that has been shown to be the key domain that binds the ACE2 receptor during entry ^44^. Administration of this domain, 193 amino acids in size, has been shown to effectively block the entry of SARS in cell culture ^44^. It is well within reason that SARS RBD could be given to patients, thereby binding their ACE2 proteins on target cells, preventing infection ( Figure 1). There is also the potential for the equivalent RBD of 2019-nCoV to be produced and used as a therapy as well. This strategy assumes SARS and 2019-nCoV share the same binding site on ACE2, which is highly likely given the similar ACE2 binding sites of SARS and NL63 coronavirus The small size of the therapy, similar in size in nanobody domains from camelid antibodies, would enhance the perfusion of the biologic into tissues to more effectively bind to viral entry receptors ^45^. In regards to the outbreak situation that is ongoing, the small protein facilitates the rapid production of the therapy in bacteria potentially, which would help production yields ^19^. Moreover, bacterial production would allow RBD proteins to be produced in a wide range of production facilities today in China, which already has numerous contract research organization operations ^46^. Alternatively, the RBD protein could be attached to an Fc fragment for extended circulation, which was done for an equivalent 212 amino acid domain from MERS. The MERS RBD-Fc fusion demonstrated the ability to block viral infection toward cell receptors, as well as to stimulate an immune response against that specific viral domain in mice ^47^. Of note, since the RBD-Fc fusion would bind to normal cells, one would want to eliminate cytotoxic Fc domain functions through mutations that eliminate Fc receptor binding ^48^.

A second, similar strategy would be to administer an antibody that would bind to ACE2 protein, thereby preventing 2019-nCoV infection ( Figure 1). This strategy was shown to effectively block SARS entry and replication in experiments ^42^. While no ACE2 antibody sequences are published in literature indexes, monoclonal antibodies do exist and the associated hybridoma sequences could be cloned in a matter of days. There would be no concern for any viral escape from an ACE2 binding antibody, which is an advantage over neutralizing approaches against the S protein. There are a couple of design considerations when thinking about how to employ the ACE2 antibody strategy. Any effector functions would need to be removed from the Fc domain ^49^, such that inflammation would not be caused in different tissues expressing ACE2. This would retain the long-half life endowed by the Fc domain without any of the side effects. The downside of including the Fc domain is the need to use a more expensive mammalian cell production system to preserve proper glycosylation, which would decrease the turnaround time for getting the drug to patients in the outbreak scenario. Alternatively, one could just administer a single chain variable fragment (scFv) that binds to ACE2. A nanobody or VHH domains from camelids are another option as well ^50, 51^. These could be produced in bacteria, and its small size would allow for rapid permeation into different tissues. The downside is the shorter half-life of these molecules without the Fc domain.

There are several limitations to these two options. Regarding the SARS RBD strategy, the body would likely develop an immune response to the SARS protein eventually, although the key intervention period of infection to combat 2019-nCoV would fall under this window of time, where after an immune response for both viruses would develop. Alternatively, if one were to use the homologous RBD from 2019-nCoV itself, this immune response would likely be very advantageous since it could yield both a blocking effect and a vaccination effect ^52^. For both strategies, the dose that would be needed to block ACE2 receptors in the body across different organs is unknown, and as is the percentage of ACE2 receptors that would need to be saturated in order to slow the infection. The number of ACE2 receptors in the body, which are found in lung and gastrointestinal organs along with vascular endothelial cells among other tissues ^53^, could ultimately prove prohibitive for this strategy. Moreover, the turnover of the ACE2 receptor on the cell surface would also influence how often the therapeutic protein would need to be administered. To solve this issue, one could increase the concentration of anti-ACE2 therapy at the crucial site of infection in the lungs, via local administration to lungs via nebulization. Lastly, there is the possibility that binding ACE2 directly could paradoxically worsen lung physiology and clinical symptoms. A study found that a fusion protein of SARS RBD to Fc domain bound ACE2 in murine lung tissue after administration, exacerbating alveolar edema via ACE2 interaction, which normally counteracts acute lung injury ^54^. This suggests that if one were use an ACE2 binding strategy, it would be best employed early during infection or as a prophylaxis to block the initial viral infection. Ultimately, clinical trials in patients would need to investigate these potential issues.

### ACE2 immunoadhesin strategy

A potentially more promising strategy would be to create an antibody-like molecule that would bind to the coronavirus itself, rather than shielding cells from being infected. For this strategy, it is proposed to use a soluble version of the ACE2 receptor that would bind to the S protein of 2019-nCoV thereby neutralizing the virus ( Figure 1). Again, the research on the SARS virus suggests this strategy is potentially promising. Soluble ACE2 receptor was demonstrated to block the SARS virus from infecting cells in culture ^42^. The reported affinity of soluble ACE2 for the SARS S protein was 1.70 nM, which is comparable to the affinities of monoclonal antibodies ^55^; it is likely that 2019-nCoV has similar affinity for ACE2. In order to use ACE2 as a therapy to treat patients, it would be advisable to convert soluble ACE2 into an immunoadhesin format fused to an immunoglobulin Fc domain (ACE2-Fc), thereby extending the lifespan of the circulating molecule, while also recruiting effector functions of the immune system against the virus. While not tested in an animal model, a previous study demonstrated that an ACE2 extracellular domain fused to the human IgG1 domain (ACE2-NN-Ig) was effective in neutralizing SARS coronavirus *in vitro*, with a 50% inhibitory concentration of 2 nM ^56^. This study provides evidence then that ACE2-Fc could similarly inhibit 2019-nCoV *in vitro* and potentially in patients.

An additional advantage of using ACE2 as a 2019-nCoV S protein neutralizing agent is that ACE2 administration could also directly treat the pneumonia pathophysiology. A portion of patients with SARS and 2019-nCoV infection develop pneumonia, which is characterized by pulmonary edema and acute respiratory distress syndrome (ARDS) ^1, 2^. The viruses may, in part, cause ARDS through viral-induced ACE2 protein shedding and ACE2 protein decreased expression, both of which are mediated by S protein binding ^54^. Administration of recombinant ACE2 protein has been shown to improve acute lung injury through decreasing angiotensin II levels and the hormones subsequent binding to angiotensin II type 1a receptor ^57^. Recombinant ACE2 can also reduce ARDS in respiratory syncytial virus ^58^ and H5N1 influenza ^59^ infection models. Based on these promising preclinical studies, recombinant human ACE2 (rhACE2) was moved into clinical trials in order to treat ARDS in critically ill patients. A phase I trial demonstrated rhACE2 was well tolerated with no effects seen on the cardiovascular system ^60^. A phase II trial demonstrated on-target efficacy in reducing Ang1-8 peptide levels, but did not show significant modulation of respiratory parameters ^61^. It remains to be seen whether rhACE2 administration has the same clinical benefits in treating ARDS that have been seen in animal models, and whether ACE2-Fc administration could alleviate ARDS in 2019-nCoV patients.

**Figure 2.**
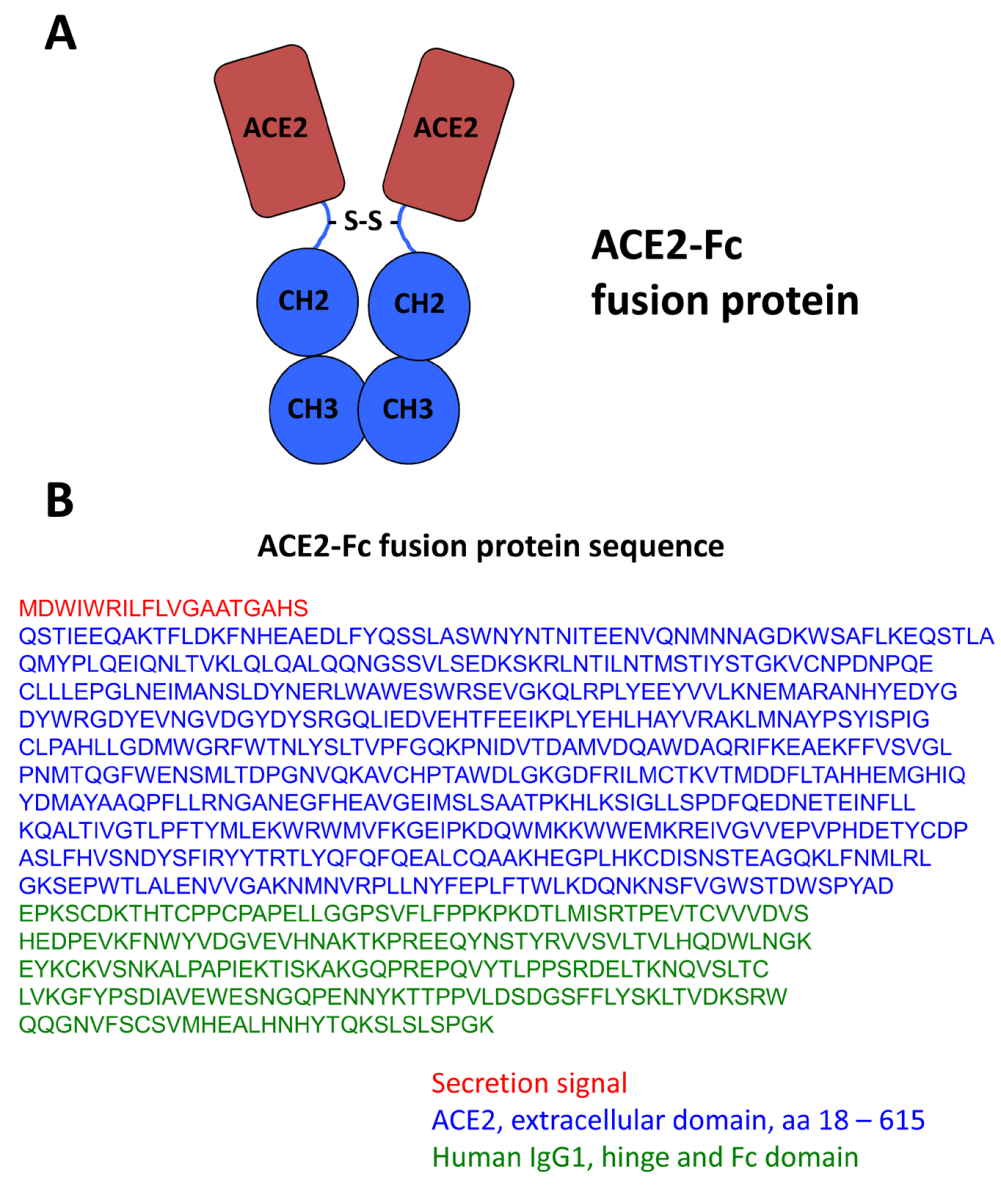
TherapeuticDesign of the ACE2-Fc fusion protein as a therapy against 2019-nCoV coronavirus. ( **A**) The extracellular domain of ACE2 is appended onto the human immunoglobulin Fc domain, including the hinge region. The Fc domain facilitates dimerization of two ACE2 domains. ( **B**) The amino acid sequence of the ACE2-Fc fusion protein is provided. The ACE2 domain consists of amino acids 18–615 of the human ACE2 protein (blue; UniProtKB - Q9BYF1). The sequence of the human immunoglobulin G isotype 1 constant region is provided (green; UniProtKB - P01857). A secretion signal from a human immunoglobin heavy chain is provided (red; UniProtKB - A0A0C4DH39).

The proposed therapy for 2019-nCoV patients would consist of the extracellular domain of the ACE2 protein fused to a human immunoglobulin G Fc domain ( Figure 2A). Studies have shown that the ACE2 amino acids 18 – 615 appear to be sufficient for SARS S protein binding ^62^, which also covers the peptidase domain necessary for ACE2 enzymatic function. It is possible a smaller portion of the extracellular ACE2 domain would be adequate for S protein binding, although a smaller version would lack enzyme activity beneficial in treating lung injury. Further studies are needed to define the minimal ACE2 domain necessary for 2019-nCoV S protein binding to construct even smaller ACE2-Fc proteins. While we do not know the structure of the 2019-nCoV S protein or how it binds to the ACE2 receptor yet, it is reasonable for now to assume that the same ACE2 protein domains utilized by the SARS virus are also bound by 2019-nCoV to infect cells.

The advantage of the Fc domain is endowing a longer-half life of the drug, which could enable healthcare workers to potentially be given drug doses prophylactically before seeing infected patients. Indeed, the half-life of recombinant ACE2 was extended from less than two hours to over one week in mice when formatted as a recombinant ACE2-Fc therapy in a study evaluating treatment for hypertension ^63^. One difference from the prior blocking agent strategies is that the effector functions of the Fc domain could be retained in this molecule, allowing recruitment of dendritic cells, macrophages, and natural killer cells through the CD16 receptor against viral particles or infected cells. This may facilitate faster activation of the host antiviral immune response and elimination of the virus, which was illustrated in a SARS mouse model where Fc engaging antibodies were more potent in eliminating SARS via activation of phagocytic cells compared to antibodies that neutralized virus alone ^64^. Overall, the ACE2-Fc fusion protein would have many of the same benefits of a traditional neutralizing antibody that would be sought as a treatment for the infection, but represent one with maximal breadth and potency since the 2019-nCoV could not escape its neutralization, given the same protein is also its receptor for cell entry. Indeed, it has been shown that the pathogenicity of SARS versus the more mild human coronavirus NL63 was related to a lower affinity of NL63 for human ACE2 versus SARS, with NL63 S protein reducing ACE2 levels less than SARS S protein ^65^. Therefore, if 2019-nCoV were to try to escape ACE2 neutralization via decreasing affinity, it would mutate into a less pathogenic virus. This is similar to the re-emergent SARS virus in 2003-2004, which had lower affinity for human ACE2 and resulted in less severe infection and no secondary transmission ^66^. Thus, 2019-nCoV could be presented with an evolutionary trap when faced with potential ACE2-Fc therapy, leading toward a more benign clinical course.

To give some additional support to the potential of a receptor-immunoadhesin being a potential antiviral strategy, it should be noted that CD4-Fc or CD4-IgG was one of the early agents developed as a potential HIV medication ^67^. The protein contained the first two domains of the CD4 receptor that are known to bind gp120 on the surface of infected HIV cells. CD4-IgG was shown to neutralize HIV *in vitro*, preventing infection. The protein was also safe when administered in patients, although only limited-to-mild clinical benefit was achieved ^68, 69^. Updated enhanced versions of CD4-IgG have been developed that additionally have a small peptide derived from the co-receptor, CCR5, enhancing affinity and giving even more potent neutralizing activity, essentially 100% of HIV isolates and making rhesus macaques resistant to multiple simian-human immunodeficiency virus challenges ^70, 71^. While HIV and 2019-nCoV are very different viruses, with different cell types, kinetics, and clinical courses, the previous results with HIV are encouraging that this could be a therapeutic strategy for 2019-nCoV. If anything, 2019-nCoV is likely more amenable to this neutralizing therapy given that the respiratory virus will only cause an acute infection, unlike HIV, which causes chronic infection in hosts with different cellular reservoirs.

One potential limitation of the ACE2-Fc strategy is that the increase in levels of extracellular ACE2 could have unknown effects on the body, particularly when elevated for a prolonged time via Fc domain extended half-life. Small levels of extracellular ACE2 are already secreted by tissues, so the circulation of this extracellular domain would not be unprecedented ^72^. Moreover, recombinant ACE2 protein was well-tolerated by healthy patients in a phase I trial, and by patients with lung injury in a phase II trial, suggesting treating 2019-nCoV patients with ACE2-Fc will also tolerated. If investigators are still concerned, critical amino acid(s) for ACE2 peptidase activity could be mutated to abolish the native function of this sequence, while retaining high affinity binding for SARS and 2019-nCoV S protein. Indeed, this possibility was previously investigated in generating an ACE2 and IgG1 fusion protein, which showed that mutation of histidine residues at position 374 and 378 of the ACE2 extracellular domain abolished peptidase activity, but retained high affinity binding to SARS S protein ^56^. Of course, ACE2 peptidase mutation would eliminate the beneficial effects from the recombinant protein delivery of ACE2 in treating lung injury, so it is recommended that retaining ACE2 enzyme activity be pursued first. Another potential concern is that receptor binding via an antibody format could inadvertently direct 2019-nCoV toward infecting Fc receptor (CD16) positive cells, which has been shown *in vitro* for neutralizing antibodies in MERS ^73^. It’s unclear what clinical significance this would have, and to what extent this would happen *in vivo*. Ultimately, clinical trials will be needed to delineate any specific side effects of ACE2-Fc treatment.

## Action plan and discussion

The chief objective of global health efforts against 2019-nCoV remains to effectively quarantine patients and screen individuals who may be infected to limit spread. That objective should continue going forward. What is proposed here is an option to at least give infected patients a medication quickly to help alleviate symptoms and prevent death, while vaccine efforts for 2019-nCoV continue. This could be enabled through ACE2-Fc providing a triple mechanism of action: (1) Treatment of ACE2 deficiency and lung injury, (2) virus neutralization, and (3) immune effector recruitment. Beyond infected patients, ACE2-Fc could provide passive immunity to healthcare works at risk as another benefit. Going forward, it is recommended that physicians, scientists, and biotech industry in China and elsewhere pursue manufacturing an ACE2-Fc biologic agent right now, which can immediately advance into trials. A variety of different protein expression platforms (CHO, insect, yeast) could be utilized, depending on the particular contract manufacturer’s expertise. Gene therapy could even be considered to make ACE2-Fc from a DNA or mRNA platform, but would have additional risk of uncertain delivery strategies and ultimately may slow down progress toward treating patients.

The goal would be that ACE2-Fc could treat infection in current patients preventing significant morbidities and death, while also serving as a potential prophylactic to give passive immunity to clinical providers on the frontlines, as well as individuals who may have been exposed to the virus. Essentially, ACE2-Fc could be the potent neutralizing antibody that the global health community needs to combat 2019-nCoV, today, while also treating the underlying ARDS pathophysiology causing patient mortality. It could be scaled much more quickly than convalescent patient sera, which would be dependent on infected individuals to make. ACE2-Fc would be resistant to viral escape as well, unlike potential neutralizing monoclonal antibodies that may be developed in the coming weeks to months.

While a therapeutic strategy is being outlined here, the long-term goal of 2019-nCoV research would remain developing an effective vaccine to yield neutralizing antibodies, likely based on the S protein and specifically, the RBD protein. Such trials should happen as soon as possible, but may prove to be challenging to get the right level of immunogenicity, antigen presentation, adjuvant addition, and potent antibody stimulation. The virus could continue mutating, foiling different efforts to stimulate protective immunity. By comparison, 2019-nCoV cannot escape the ACE2-Fc treatment strategy, since it leverages its own cognate receptor for infection. As mentioned above, should 2019-nCoV attempt to escape this therapy via reduced ACE2 affinity binding, it would likely become less pathogenic, similar to the comparison of SARS versus human coronavirus NL63 ^65^. Lastly, scaling the dose of any effective vaccine would also prove to be challenging depending on the vector format (e.g. viral vector versus mRNA versus protein), and even a fully protective vaccine would not help patients who are currently infected with the virus.

In an effort to help aide researchers and industry in China to combat 2019-nCoV, the protein sequence of the ACE2-Fc construct is provided ( Figure 2B). Different human Fc domains (IgG1, IgG2, IgG3, or IgG4) could be contemplated, although IgG1 traditionally has the most potency for triggering anti-microbial responses ^49^. Similarly, an ACE2-Fc biologic without active ACE2 peptidase function could be explored as well. Given that gene synthesis of this sequence could happen within a week, the gene could be placed within the protein expression platform of choice shortly thereafter leading to protein production quickly. The availability of protein A columns and other techniques in the industry to purify antibodies would facilitate ACE2-Fc to quickly be repurposed on existing antibody manufacturing infrastructure existing in China.

A final benefit of pursuing ACE2-Fc is that it could effectively be used as a therapeutic drug stockpile for future outbreaks of SARS and 2019-nCoV, and any new coronavirus that emerges from a zoonotic reservoir in the future that uses the ACE2 receptor for entry. Moreover, coronaviruses that replicate in animals across China and other countries could be studied in order to assess their entry mechanisms. By understanding entry in these other animals, one could effectively predict a receptor that could be utilized in any zoonotic transmission event, and build a new receptor immunoadhesin molecule in the future. As an example, a similar immunoadhesin, DPP4-Fc, could be envisioned for MERS based on the viral receptor, DPP4, used by that virus ^74^. Beyond coronaviruses, this strategy could be utilized for other viruses where the risk of outbreak potential is high. ACE2-Fc could also find use in treating ARDS for other unrelated viruses and causes of acute lung injury, building on the previous clinical trial work ^60, 61^.

In summary, ACE2-Fc has the potential to be the neutralizing antibody that healthcare workers need to treat and prevent 2019-nCoV infection today and could play an important role in the cessation of the outbreak if manufacturing based on an available sequence starts soon. An alternative 2019-nCoV RBD-Fc fusion could also be pursued, if one desired the dual function of receptor blocking and vaccination in one molecule.

## Data availability

### Underlying data

No data are associated with this article.
