# Supplementary material for: Therapeutic strategies in an outbreak scenario to treat the novel coronavirus originating in Wuhan, China
Source: F1000Res. 2020 Feb 7;9:72. Originally published 2020 Jan 31. [Version 2] doi: 10.12688/f1000research.22211.2 (PMC7029759; doi:10.12688/f1000research.22211.2)
Supplement: Peer review history

# Peer review: Reviewer response for version 2

**DOI:** 10.5256/f1000research.24634.r59434

**Published:** 2020-02-18

**Authors:** Michael Farzan, Huihui Mou

**Affiliations:** 1 Department of Immunology and Microbiology, The Scripps Research Institute, Jupiter, FL, USA

A very sensible opinion article based on information well known to the coronavirus field but perhaps not well appreciated by others who might want to address this potential pandemic. 

 I found no factual or interpretive errors. Were the issue not so pressing, I might have somewhat discounted the article based on 'obviousness' but this is obviousness to the field and not to the wider group of readers.

We confirm that we have read this submission and believe that we have an appropriate level of expertise to confirm that it is of an acceptable scientific standard.

# Peer review: Reviewer response for version 2

**DOI:** 10.5256/f1000research.24634.r59692

**Published:** 2020-02-11

**Authors:** Yi-Wei Tang

**Affiliations:** 1 Cepheid, Danaher Diagnostic Platform, Shanghai, China

This review provide an authoritative review on 2019-nCoV infection therapeutic options. After briefly covered several treatment strategies, the author focused on options to block virus from entering cells. Figure 1 is terrific.

1. I suggest that the authors provide a table contrasting the four strategies mentioned at the beginning.
2. In the treatment involving neutralizing antibodies, the author should discuss potential side effect such as antibody dependent enhancement (ADE).
3. So many therapeutic options have been mentioned in China including Chinese herbs and chlorquine. It will be great if the author can briefly review and comment on this.

I confirm that I have read this submission and believe that I have an appropriate level of expertise to confirm that it is of an acceptable scientific standard.
